# Efficacy Comparison of Intravitreal Anti-VEGF Therapy for Three Subtypes of Neovascular Age-Related Macular Degeneration: A Systematic Review and Meta-Analysis

**DOI:** 10.1155/2018/1425707

**Published:** 2018-10-23

**Authors:** Jianqing Li, Jiayi Xu, Yiyi Chen, Jiaju Zhang, Yihong Cao, Peirong Lu

**Affiliations:** Department of Ophthalmology, The First Affiliated Hospital of Soochow University, 188 Shizi Street, Suzhou 215006, China

## Abstract

**Purpose:**

Intravitreal antivascular endothelial growth factor (anti-VEGF) therapy has been widely used for the treatment of neovascularization (NV) secondary to age-related macular degeneration (AMD). This study aimed to compare the efficacy among different subtypes of neovascular age-related macular degeneration (nAMD).

**Methods:**

PubMed, Embase, and the Cochrane Library were searched for eligible studies. We performed meta-analysis using Review Manager 5.3 and Stata/SE 12.0.

**Results:**

A total of 24 studies met our inclusion criteria and were included in the systematic review. At 3 months, the mean logarithm of the minimum angle of resolution (logMAR) improvements were −0.09, −0.18, and −0.23 for type 1, 2, and 3, respectively, while the mean macular thickness (MT) changes were −104.83, −130.76, and −196.29 *μ*m. At 12 months, the mean changes in Early Treatment of Diabetic Retinopathy Study (ETDRS) letters were 6.38, 8.12, and 9.37, while the MT decrease was 126.51, 126.52, and 139.85 *μ*m, respectively. However, statistically significant difference was only found between type 1 and 3 in vision improvement, both in the short term (*p*=0.0002) and long term (*p*=0.01).

**Conclusions:**

The reactivity to VEGF inhibitors varied among different subtypes of nAMD. The efficacy of intravitreal anti-VEGF therapy in type 3 nAMD was statistically better than type 1 when considering vision improvement at 3 and 12 months. Thus, the lesion subtype is a predictor for the treatment outcome which can help guide prognosis.

## 1. Introduction

Age-related macular degeneration (AMD) is a progressive chronic disease of the central retina and a leading cause of vision loss worldwide [1] which basically has two types: exudative, neovascular, or wet AMD and nonexudative or dry AMD [2]. Neovascular age-related macular degeneration (nAMD), characterized by aberrant angiogenesis originating from the choroidal or, less frequently, the retinal circulation [3], is responsible for nearly 90% of the severe central visual acuity loss associated with AMD despite its lower incidence compared with the dry form [4].

The classification of nAMD was first developed in 1991 [5], which was based on fluorescein angiography (FA) and characterized lesion as “classic” or well-defined choroidal neovascularization (CNV) and “occult” or poorly defined CNV. A histologic classification proposed by Gass in 1994 [6] contained two different types: type 1 (located beneath the retinal pigment epithelium (RPE)) and type 2 (present beneath the sensory retina). Additional subtypes of nAMD, such as polypoidal choroidal vasculopathy (PCV) and retinal angiomatous proliferation (RAP), were further detailed with the development of optical coherence tomography (OCT). With advancements in imaging, a new anatomic classification based on FA and OCT was proposed [7], categorizing lesions as type 1 (sub-RPE), type 2 (subretinal), type 3 (intraretinal), or mixed neovascularization (NV). PCV was considered to be a special form of type 1 nAMD [7], while occult, classic, and RAP corresponded to type 1, 2, and 3, respectively, [8] in our meta-analysis.

Intravitreal antivascular endothelial growth factor (anti-VEGF) therapy has been identified to possess the potential to stabilize or even improve visual acuity in nAMD by clinical trials [9]. Several articles have studied the efficacy of anti-VEGF on different subtypes of nAMD [10–12]; however, there has been no meta-analysis focusing on the efficacy comparison among all three types of nAMD. Therefore, we carried out a systematic review and meta-analysis in order to study the relationship between treatment efficacy and lesion subtype.

## 2. Materials and Methods

### 2.1. Search Strategy

Two independent reviewers (J. Li and J. Xu) performed a systematic search in PubMed, Embase, and the Cochrane Library on March 16, 2018, for articles focusing on the efficacy of intravitreal anti-VEGF on different subtypes of nAMD. The search strategy was [(“nAMD” AND “subtype”) AND “anti-VEGF”] using the MeSH or Emtree terms as well as free words. Here, “nAMD,” “wet AMD,” and “exudative AMD” were searched for “nAMD”; “Type 1,” “PCV,” “occult,” “poorly-defined,” “sub-RPE,” “Type 2,” “classic,” “well-defined,” “subretinal,” “Type 3,” “RAP,” and “intraretinal” were for different subtypes of nAMD, while “pegaptanib,” “bevacizumab,” “ranibizumab,” “aflibercept” and “conbercept” were searched for “anti-VEGF.”

### 2.2. Selection Criteria

The eligibility criteria were as follows: (1) any subtype of nAMD, (2) treatment-naive nAMD, (3) anti-VEGF monotherapy, (4) efficacy measured by vision improvement or macular thickness (MT) changes, and (5) efficacy measured at 3 months or 12 months. Those studies which were not in English with no full text or irrelevant data were excluded. Any disagreements about the inclusion of an article for full review were resolved by a third researcher (P. Lu).

### 2.3. Assessment of Risk of Bias

“Risk of bias” of each included article was assessed using “risk of bias table,” which was suitable for both randomized and nonrandomized studies, according to the Cochrane Handbook for Systematic Reviews of Interventions Version 5.1.

### 2.4. Data Extraction

The characteristics extracted from the eligible articles included the first author's name, publication year, country where the study was conducted, subtype of nAMD, sample size, gender, mean age of the sample, vision criteria for recruitment, anti-VEGF use, and study design. Besides, PCV was marked in the column of subtype of nAMD in Table 1 since it was a variant of type 1 nAMD.

The main outcome of this meta-analysis was efficacy comparison of intravitreal anti-VEGF therapy for three subtypes of nAMD. The efficacy was measured by vision improvement or macular thickness (MT) changes at 3 or 12 months; therefore, these data were extracted for further analysis. Since meta-analysis was a second source, the relevant data were extracted either directly from the article or by extrapolation. In this study, no authors were contacted for the raw data of each patient, thus we could not adjust some different factors such as visual acuity at baseline.

### 2.5. Statistical Analysis

All the statistical analyses in our study were completed using Review Manager (RevMan) version 5.3 (The Nordic Cochrane Centre, Copenhagen, Denmark) and Stata version 12.0 (StataCorp, Texas, America). The effect value in this meta-analysis was the mean difference (MD). Before the analysis, the study heterogeneity was tested using both the I-squared and chi-squared test statistics. An *I*^2^ ≥ 50% and/or a *Q*-statistic of *p*<0.05 was evidence supporting the presence of heterogeneity, in which the random-effects modeling method was needed. Otherwise, the fixed-effects modeling method was applied. In addition, publication bias and sensitivity analysis were conducted to study the relevant bias as well as stability and reliability of the outcomes.

## 3. Results

### 3.1. Description of Studies

**Figure 1:**
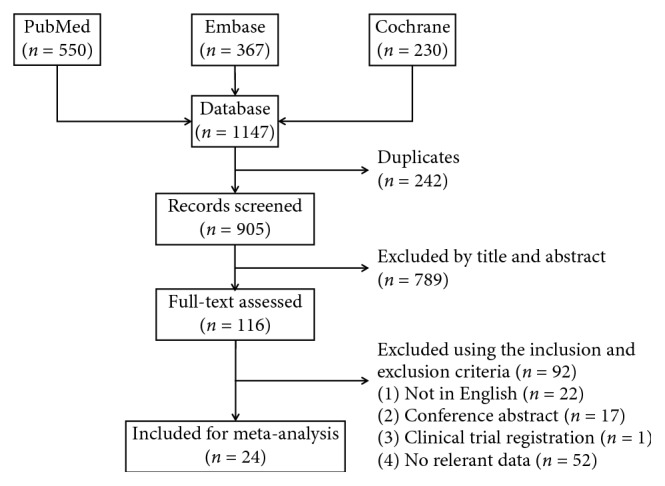
Flow diagram of the inclusion of studies in this meta-analysis.

A total of 1147 articles were identified, and their records were included in EndNote X8 (Clarivate Analytics, Philadelphia, PA, US). After removing 242 duplicates, the remaining 905 articles were screened based on the titles and abstracts by two reviewers according to our inclusion criteria. Any disagreements about the inclusion of an article for full review were resolved by the third researcher. Full-text assessment was conducted on the rest of the 116 articles. Finally, 24 articles [13–36] were included in this meta-analysis. The article searches and selection process is summarized in Figure 1.

### 3.2. Study Quality and Characteristics

**Figure 2:**
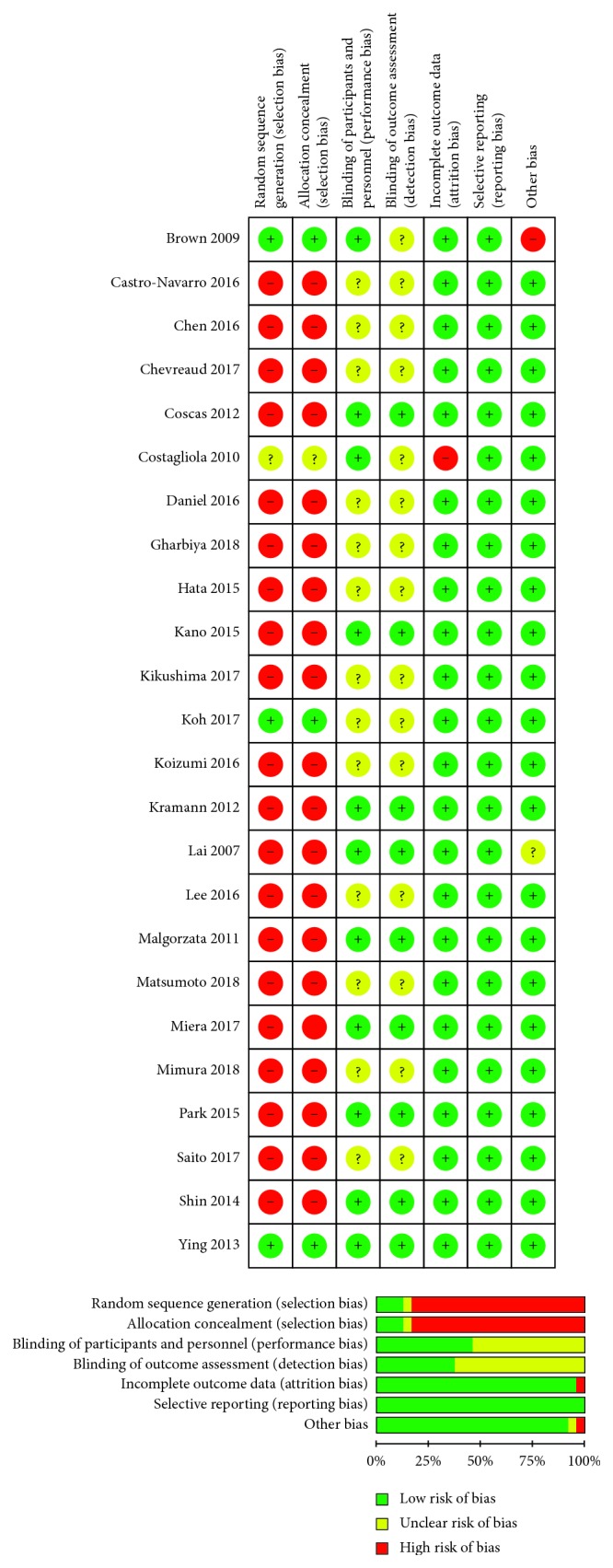
Risk of bias assessment of the included studies.

“Risk of bias” of these studies was assessed by “risk of bias table,” and the outcome is summarized in Figure 2. The selection bias, which contained random sequence generation and allocation concealment, was mainly of high risk, predominantly due to those nonrandomized studies. Although blinding of performance and detection was not performed in most of the included studies, the outcome was not likely to be influenced by lack of blinding or there was insufficient information to permit judgement of “Low risk” or “High risk.”

Among the included studies, 4 were randomized controlled trials (RCT), 12 were retrospective interventional studies (RIS), 6 were clinical trials, and the left 2 were case reports. Altogether, 2594 patients were involved in this review. The demographic characteristics of the studies were summarized in Table 1.

### 3.3. Critical Appraisal Tool

#### 3.3.1. Short-Term Outcome

**Figure 3:**
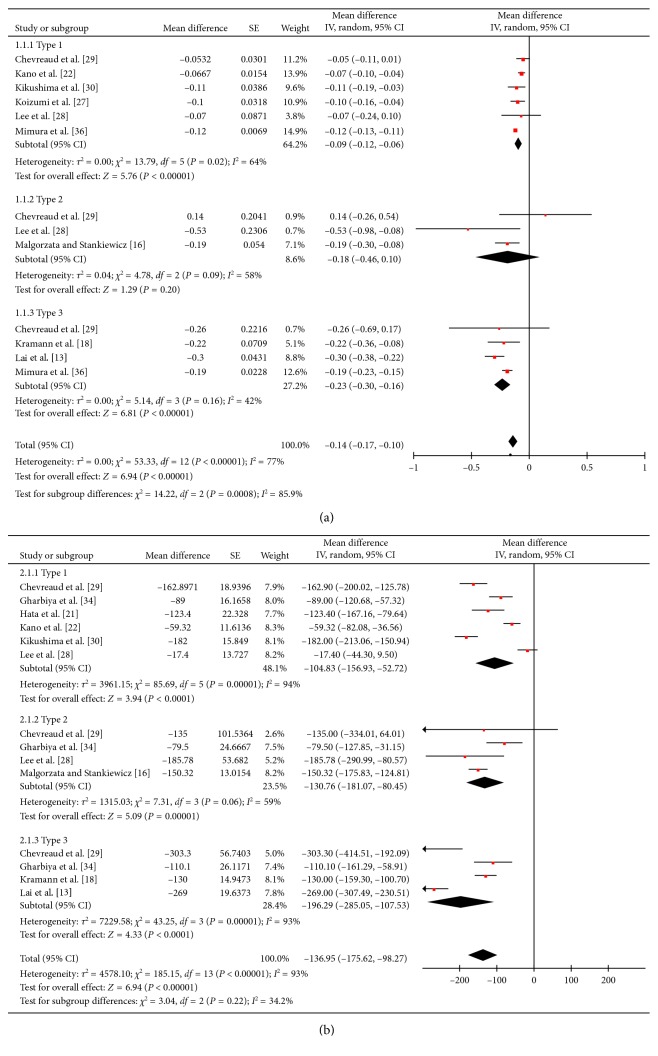
The short-term (3 months) efficacy comparison of antivascular endothelial growth factor (anti-VEGF) therapy for three subtypes of neovascular age-related macular degeneration (nAMD). (a) Vision improvement of the three types of nAMD, measured by logarithm of the minimum angle of resolution (logMAR), were −0.09 (95% confidence interval (CI): −0.12, −0.06), −0.18 (95% CI: −0.46, 0.10), and −0.23 (95% CI: −0.30, −0.16). (b) Macular thickness decreases were −104.83 (95% CI: −156.93, −52.72), −130.76 (95% CI: −181.07, −80.45), and −196.29 (95% CI: −285.05, −107.53) *μ*m, respectively.

Figure 3 illustrated the short-term (3 months) efficacy comparison. In Figure 3(a), vision improvements of the three types of nAMD, measured by logarithm of the minimum angle of resolution (logMAR), were −0.09 (95% confidence interval (CI): −0.12, −0.06), −0.18 (95% CI: −0.46, 0.10), and −0.23 (95% CI: −0.30, −0.16). While MT changes, displayed in Figure 3(b), were −104.83 (95% CI: −156.93, −52.72), −130.76 (95% CI: −181.07, −80.45), and −196.29 (95% CI: −285.05, −107.53) *μ*m, respectively. It was obvious that the efficacy varied among different subtypes of nAMD; however, the subtype difference was only statistically significant between type 1 and type 3 in vision improvement (*p*=0.0002).

#### 3.3.2. Long-Term Outcome

**Figure 4:**
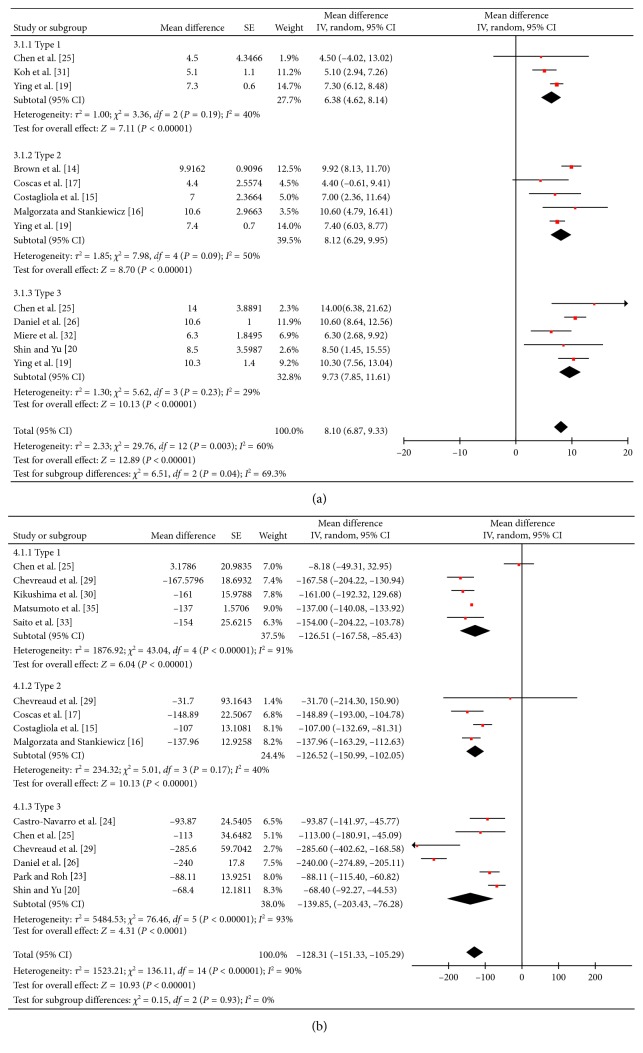
The long-term (12 months) efficacy comparison among three subtypes of neovascular age-related macular degeneration (nAMD) was presented. (a) Vision improvement, evaluated by Early Treatment of Diabetic Retinopathy Study (ETDRS) letters, were 6.38 (95% CI: 4.62, 8.14), 8.12 (95% CI: 6.29, 9.95), and 9.73 (95% CI: 7.85, 11.61) for the three types of nAMD. (b) Macular thickness changes were −126.51 (95% CI: −167.58, −85.43), −126.52 (95% CI: −150.99, −102.05), and −139.85 (95% CI: −203.43, −76.28) *μ*m, respectively.

The long-term (12 months) efficacy comparison was presented in Figure 4. Vision improvements, evaluated by Early Treatment of Diabetic Retinopathy Study (ETDRS) letters, were 6.38 (95% CI: 4.62, 8.14), 8.12 (95% CI: 6.29, 9.95), and 9.73 (95% CI: 7.85, 11, 61) for the three types of nAMD. In Figure 4(b), MT changes were −126.51 (95% CI: −167.58, −85.43), −126.52 (95% CI: −150.99, −102.05), and −139.85 (95% CI: −203.43, −76.28) *μ*m, respectively. Although the efficacy differed in the three types of nAMD, the statistically significant subtype difference still only existed between type 1 and type 3 in vision improvement (*p*<0.0001).

### 3.4. Publication Bias

**Figure 5:**
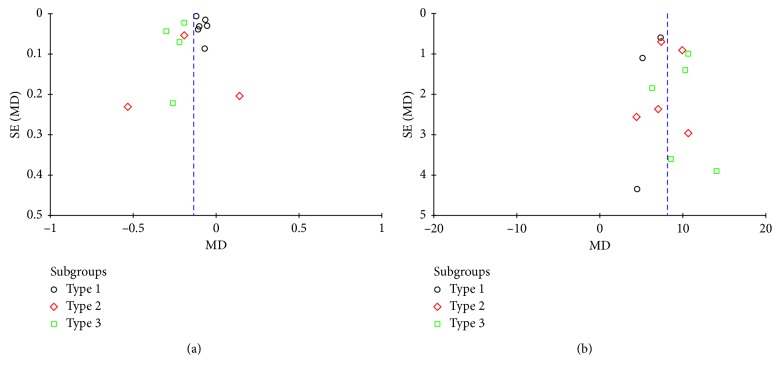
Funnel plots for short-term (a) and long-term (b) vision improvement comparison.

Funnel plots for short-term and long-term vision improvement comparison are shown in Figure 5. The two plots were both relatively symmetrical, which indicated little evidence of publication bias.

### 3.5. Sensitivity Analysis

**Figure 6:**
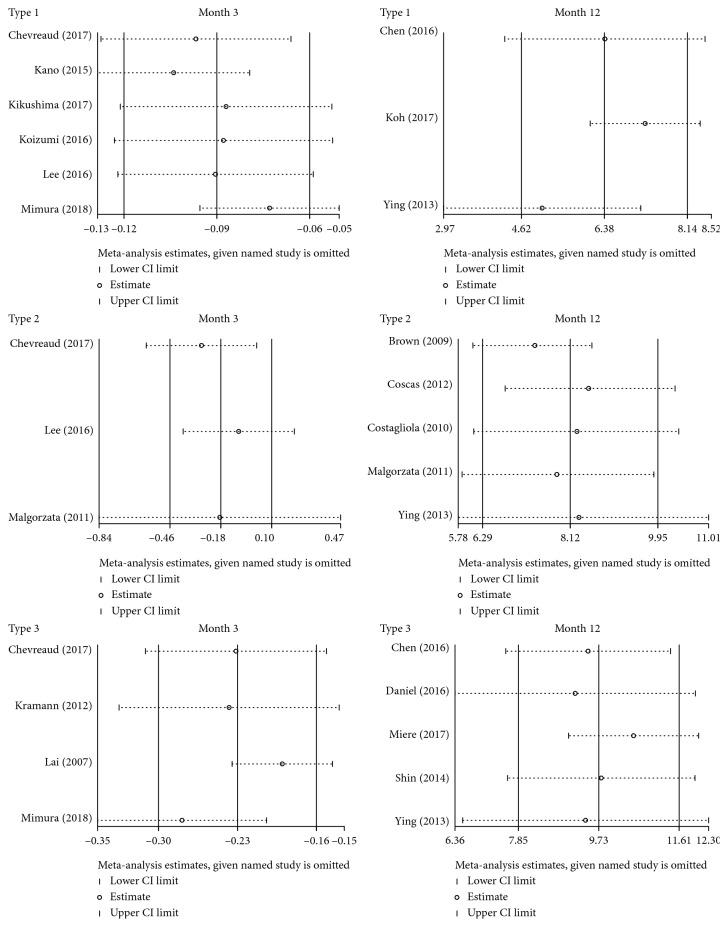
Sensitivity analysis on the outcome of vision improvement at 3 and 12 months.

The impact of individual data was examined for sensitivity analysis, which was conducted on the outcome of vision improvement at 3 and 12 months (Figure 6). When omitting each study, we found no obvious changes to the results, thus drawing a conclusion that our results on vision efficacy were stable and reliable.

## 4. Discussion

To our knowledge, this was the first meta-analysis quantitatively comparing treatment efficacy of intravitreal anti-VEGF therapy for three subtypes of nAMD. A total of 24 articles were included in our systematic review. In order to assess the efficacy, the relevant data about vision improvement and MT changes at 3 months or 12 months were extracted for further pooled analysis. We found that although the efficacy of type 2 nAMD was superior to type 1 and inferior to type 3, statistically significant difference was only found between type 1 and 3 in vision improvement, both during the short term (*p*=0.0002) and long term (*p*=0.01). We could draw a conclusion that the reactivity to VEGF inhibitors varied among different subtypes of nAMD. Thus, the lesion subtype could be considered as a predictor for the treatment outcome.

There are several potential explanations for our results. First of all, the three types of nAMD are characterized with different locations of NV: sub-RPE, subretina, and intraretina. The closer to the retinal photoreceptors, the earlier and more severe the clinical symptoms of nAMD appear. Therefore, the three subtypes suffer from different durations and various severities. Another theory is anti-VEGF resistance, which means that tissues treated with anti-VEGF may develop resistance to hypoxia and become less dependent on angiogenesis or develop more mature vessels through remodeling, which is less responsive to antiangiogenic therapy [37]. Patients with type 1 nAMD tend to agonize over longer duration, thus are more likely to develop anti-VEGF resistance. Therefore, treatment efficacy for type 1 is inferior to type 2 and 3.

So far, the three types of nAMD have been classified based on the location of abnormal vessels using fluorescence angiography (FA) and optical coherence tomography (OCT). Nevertheless, OCTA (and a possible OCTA classification) may offer additional information on treatment-response and anti-VEGF resistance by providing morphological information and quantitative measures (area, vascular density, fractal dimension, etc.), but that the clinical relevance of such findings is still debated [32, 38, 39].

Some limitations did exist in our systematic review. Firstly, this meta-analysis consisted mostly of nonrandomized studies which were more likely to be influenced by various kinds of biases. Hence, it was not surprising that almost all of them were at relatively high risk when we assessed the risk of bias as shown in Figure 2. However, there has been no way to control these biases and no method to assess their impact on the outcome so far. Secondly, heterogeneity was statistically significant across several results (Figures 3 and 4). One possible explanation was that nonrandomized studies were more likely to introduce heterogeneity due to confounding factors and all kinds of biases. Moreover, no authors were contacted for primary statistics. The relevant data were extracted either directly from the article or by extrapolation, thus we could not adjust some different factors at baseline. For example, of the 24 included studies, only 11 were involved with vision criteria for enrollment, and these criteria were various. However, we had no way to adjust visual acuity at baseline because of lacking individual patient data. Nevertheless, this problem might be a common limitation of meta-analysis.

## 5. Conclusion

In summary, we believe that this meta-analysis, comparing treatment efficacy among different types of nAMD, is of great significance to clinical practice. The results indicate that lesion subtype is a predictor for the treatment outcome which could help guide prognosis.

## Figures and Tables

**Table 1:** Baseline characteristics of the included studies.

First author (year)	Country	Subtype of nAMD	Sample size	Male (%)	Age (year)	Vision criteria for recruitment	Anti-VEGF use	Study design
Lai et al. (2007) [13]	China	Type 3	4	25.00	81.0 ± 4.12	NA	Ranibizumab	Case report
Brown et al. (2009) [14]	America	Type 2	280	52.86	76.7 ± 8.08	NA	Ranibizuma	RCT
Costagliola et al. (2010) [15]	Italy	Type 2	45	44.44	65.3 ± 15	NA	Bevacizumab	RCT
Malgorzata and Stankiewicz (2011) [16]	Poland	Type 2	25	44.00	73.23 ± 8.55	22–76 ETDRS letters	Ranibizumab	Clinical trial
Coscas et al. (2012) [17]	France	Type 2	29	34.48	76.3 ± 10.9	20/400–20/40 by the ETDRS charts	Ranibizumab	RIS
Kramann et al. (2012) [18]	Germany	Type 3	26	30.77	77 ± 8.25	NA	Ranibizumab	Retrospective case Series
Ying et al. (2013) [19]	America	Type 1, type 2, type 3	1105	38	79 ± 8	20/25–20/320 on electronic VA testing	Ranibizumab or bevacizumab	RCT
Shin and Yu (2014) [20]	South Korea	Type 3	31	19.35	70.4 ± 6.5	5–75 ETDRS letters (Snellen 20/32–20/800)	Ranibizumab	Clinical trial
Hata et al. (2015) [21]	Japan	Type 1 (PCV)	70	81.43	72.2 ± 8.8	0.7 or less on a Landolt chart	Ranibizumab	RIS
Kano et al. (2015) [22]	Japan	Type 1	100	86	75.71 ± 7.79	0.05 or better by the Japanese decimal VA chart	Ranibizumab or aflibercept	RIS
Park and Roh (2015) [23]	South Korea	Type 3	40	39.02	67.09 ± 11.76	NA	Ranibizumab	RIS
Castro-Navarro et al. (2016) [24]	Spain	Type 3	7	14.29	79.42 ± 7.14	<0.1 logMAR	Aflibercept	RIS
Chen et al. (2016) [25]	America	Type 1, type 3	36	36.11	80 ± 8.0	19–73 ETDRS letters (Snellen 20/35–20/400)	Aflibercept	Clinical trial
Daniel et al. (2016) [26]	America	Type 3	126	30.95	81.7 ± 7.30	20/25–20/320	Ranibizumab or bevacizumab	Clinical trial
Koizumi et al. (2016) [27]	Japan	Type 1 (PCV)	86	NA	NA	NA	Aflibercept	RIS
Lee et al. (2016) [28]	South Korea	Type 1, type 2	23	60.87	66.52 ± 9.28	NA	Ranibizumab	RIS
Chevreaud et al. (2017) [29]	France	Type 1, PCV, type 2, type 3	109	33.03	76.9 ± 8.3	NA	Ranibizumab	RIS
Kikushima et al. (2017) [30]	Japan	Type 1 (PCV)	69	83.9	72.9 ± 7.9	Decimal BCVA ≤ 1.2 in the Landolt chart	Aflibercept	RIS
Koh et al. (2017) [31]	Singapore	Type 1 (PCV)	154	75.3	68.2 ± 9.0	78–24 ETDRS letters (Snellen 20/32–20/320)	Ranibizumab	RCT
Miere et al. (2017) [32]	Italy	Type 3	15	33.33	82.3 ± 4.9	NA	Ranibizumab or bevacizumab	RIS
Saito et al. (2017) [33]	Japan	Type 1 (PCV)	20	90.00	72.2 ± 6.4	NA	Aflibercept	Clinical trial
Gharbiya et al. (2018) [34]	Italy	Type 1, type 2, type 3	76	34.21	78.67 ± 8.04	NA	Ranibizumab or aflibercept	Clinical trial
Matsumoto et al. (2018) [35]	Japan	Type 1 (half with PCV)	60	85.00	75.1 ± 1.0	NA	Aflibercept	RIS
Mimura et al. (2018) [36]	Japan	Type 1 (PCV), type 3	58	65.52	73.08	NA	Aflibercept	RIS

^†^Data are mean ± standard deviation or mean. ^‡^nAMD: neovascular age-related macular degeneration; anti-VEGF: antivascular endothelial growth factor; PCV: polypoidal choroidal vasculopathy; NA: not available; ETDRS: Early Treatment of Diabetic Retinopathy Study; RCT: randomized controlled trial; RIS: retrospective interventional study.
